# Supplementary material for: The Prognostic Value of ASPHD1 and ZBTB12 in Colorectal Cancer: A Machine Learning-Based Integrated Bioinformatics Approach
Source: Cancers (Basel). 2023 Aug 28;15(17):4300. doi: 10.3390/cancers15174300 (PMC10486397; doi:10.3390/cancers15174300)
Supplement: Supplementary file 1 [file cancers-15-04300-s001.zip › cancers-2564797-supplementary.pdf]

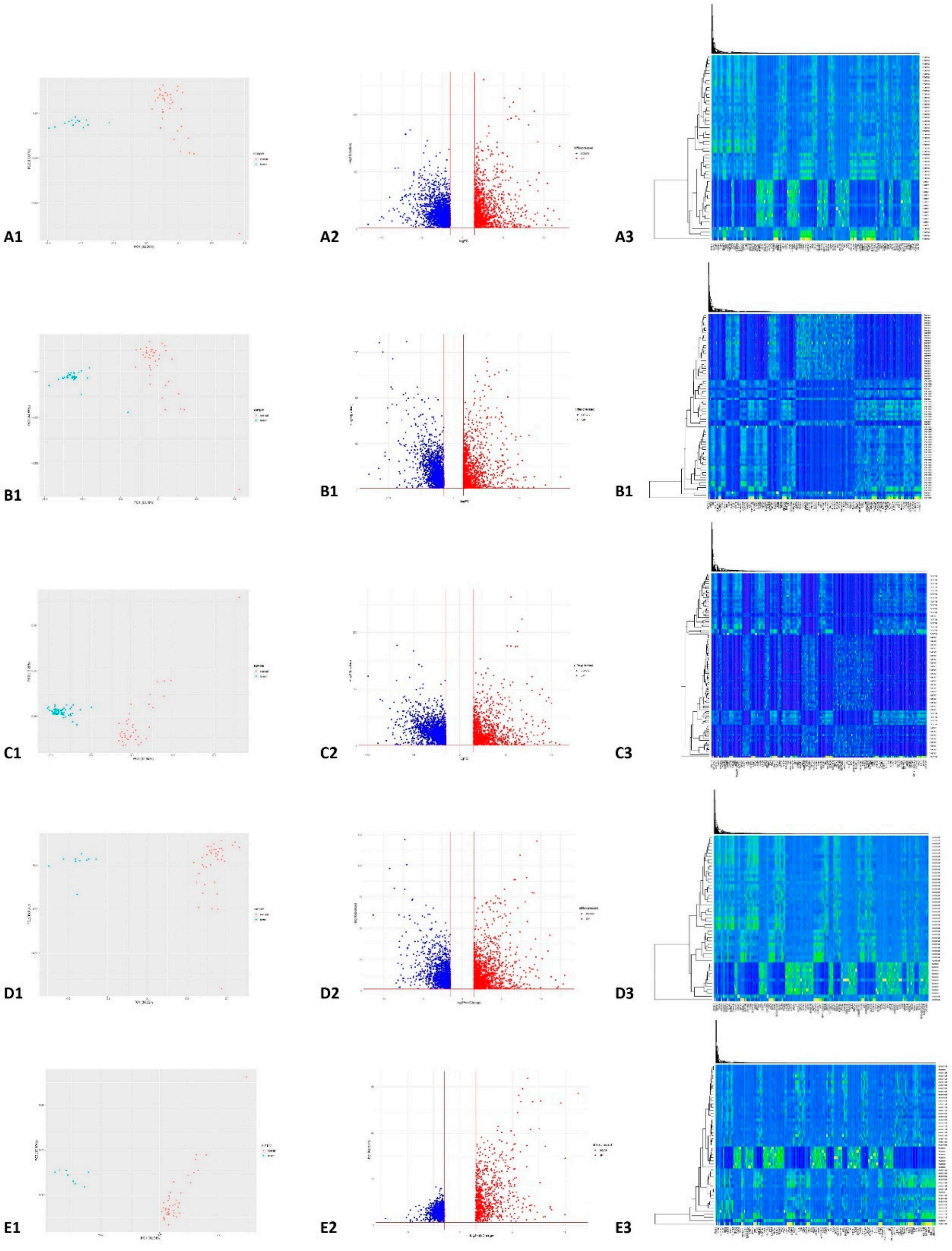

Figure S1. PCA plots, volcano plots, and heat maps for DEGs in each subgroup of CRC patients from the TCGA database. (A: MSI-L, B: MSI-H, C: MSI-S, D: Receiving chemotherapies, E: Receiving targeted therapies)

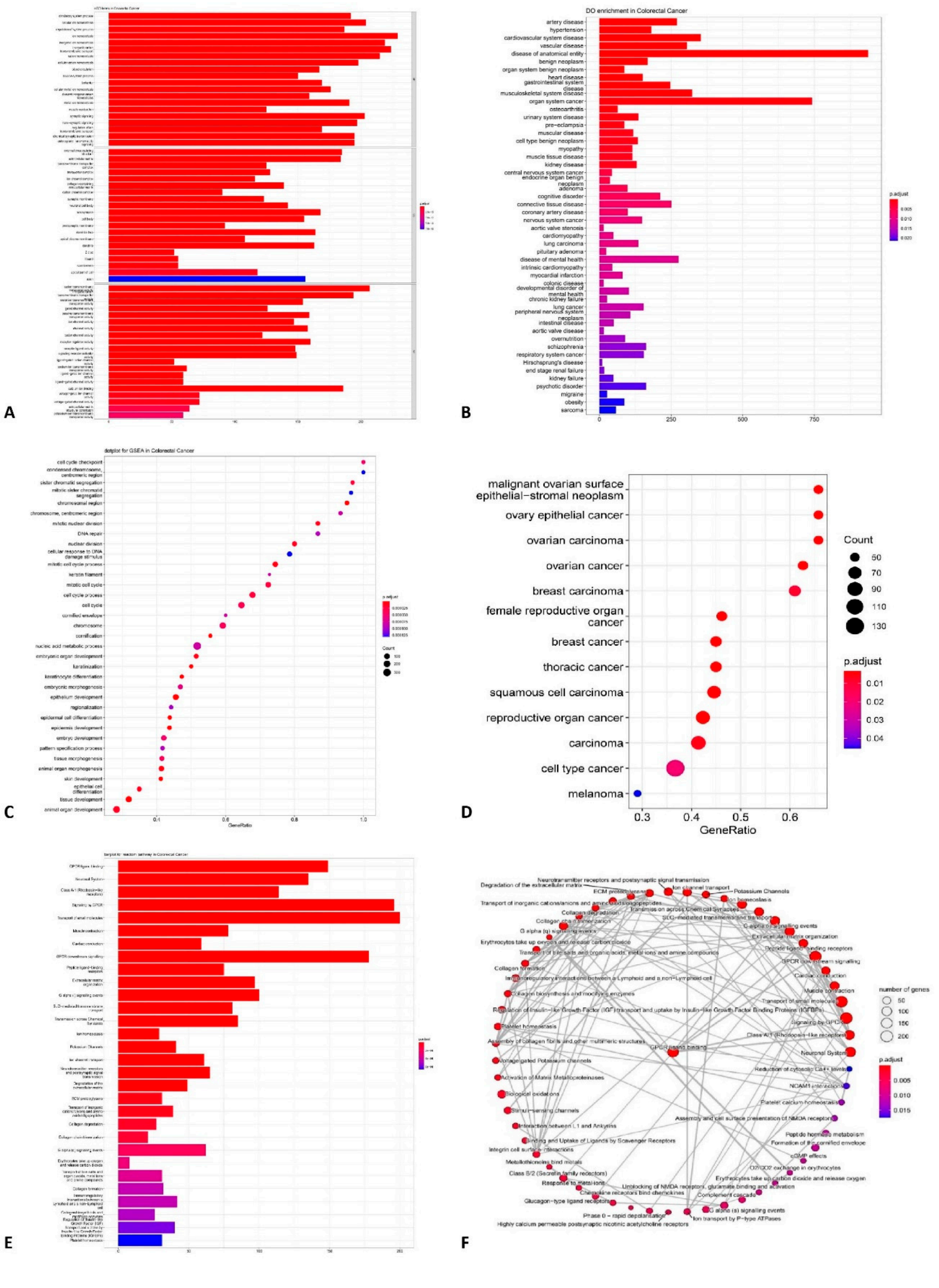

Figure S2. Pathway enrichment analyses of DEGs in MSI-L CRC patients from the TCGA database. (A: Bar plot of Gene Ontology, B: Bar plot of Disease Ontology, C and D: Dot plots for gene set enrichment analysis, E: Bar plot of Reactom, F: Network plot of signaling pathways based on Reactom)

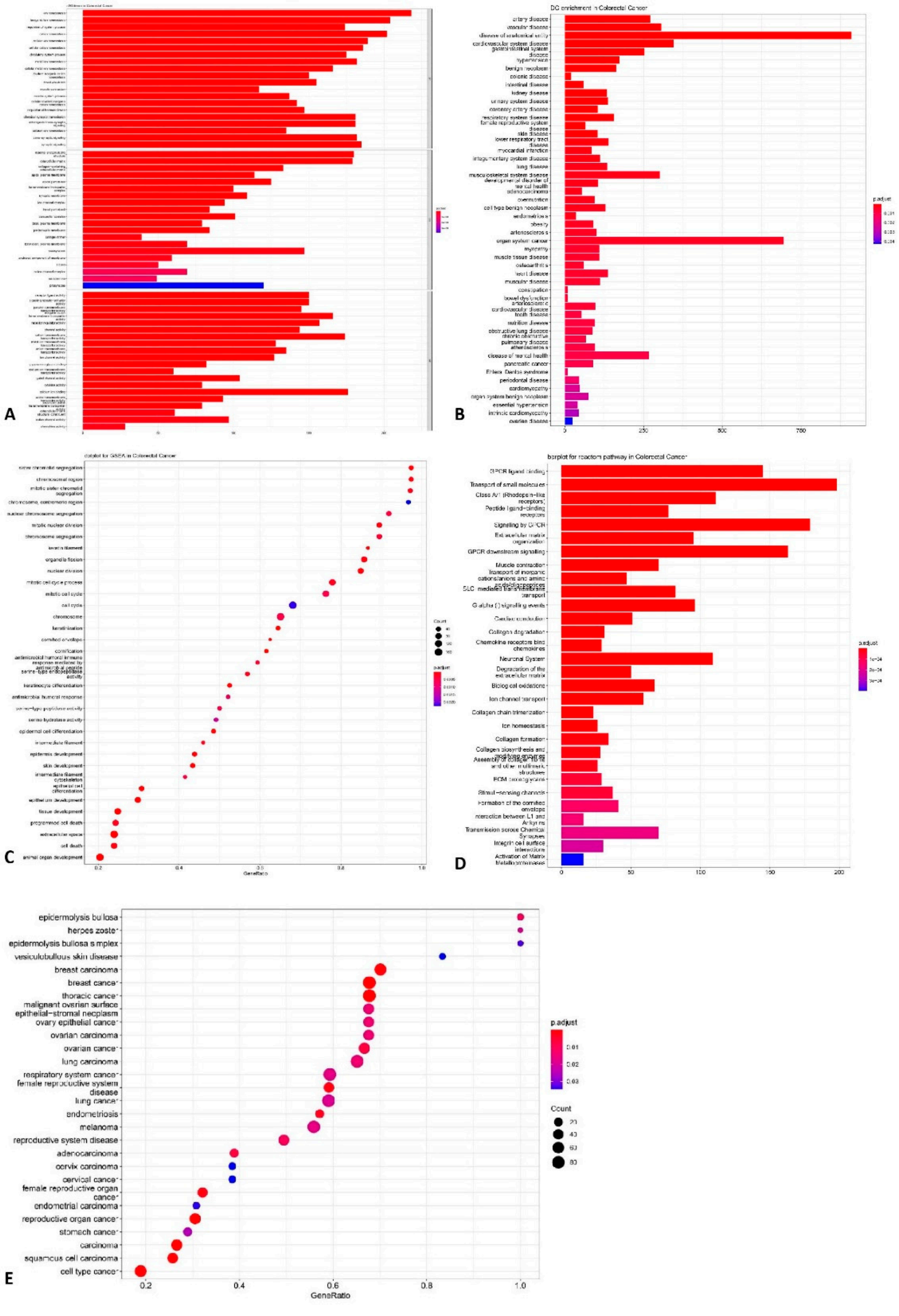

Figure S3. Pathway enrichment analyses of DEGs in MSI-H CRC patients from the TCGA database. (A: Bar plot of Gene Ontology, B: Bar plot of Disease Ontology, C and D: Dot plots for gene set enrichment analysis, E: Bar plot of Reactom)

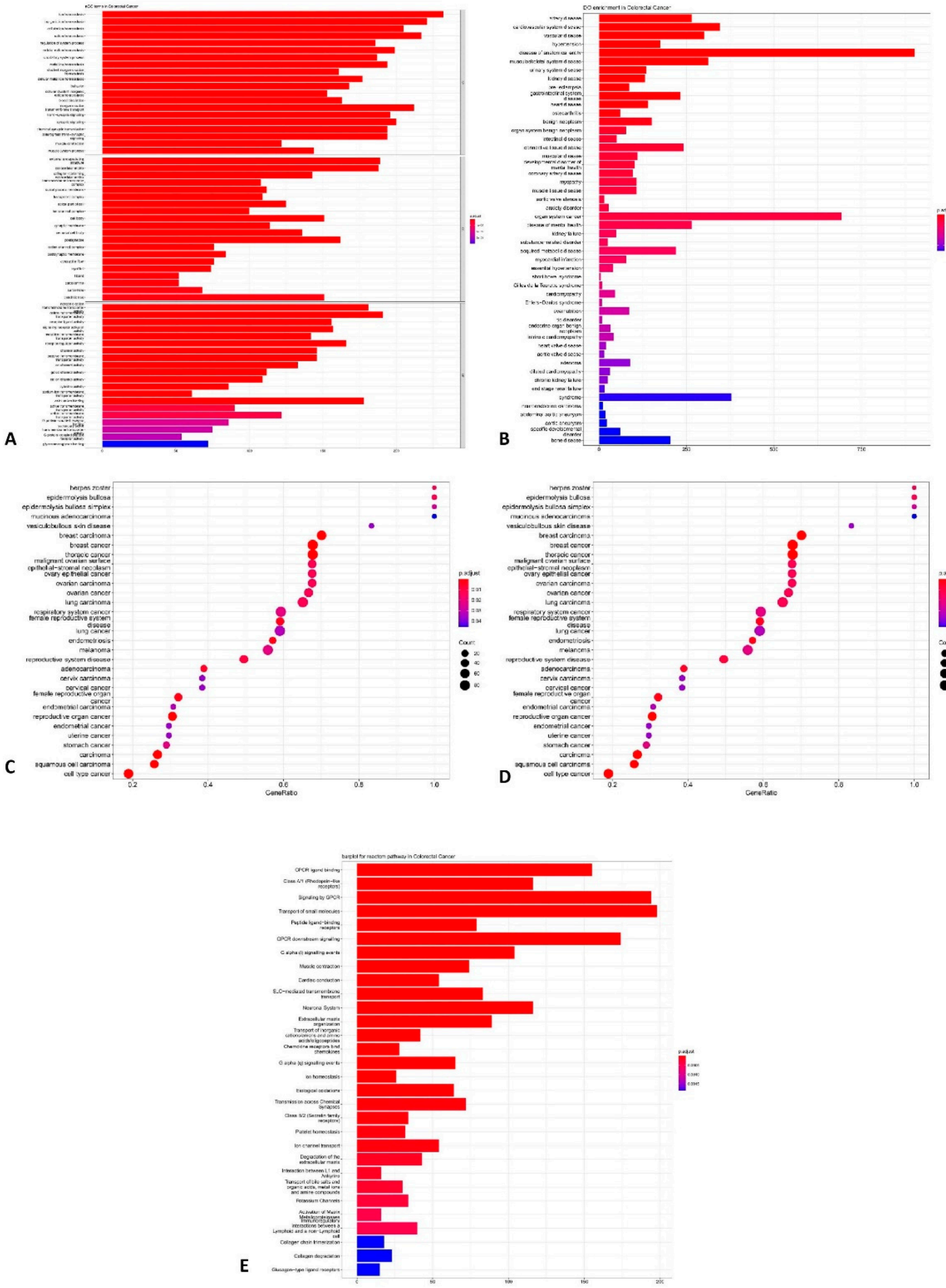

Figure S4. Pathway enrichment analyses of DEGs in MSS CRC patients from the TCGA database. (A: Bar plot of Gene Ontology, B: Bar plot of Disease Ontology, C and D: Dot plots for gene set enrichment analysis, E: Bar plot of Reactom)

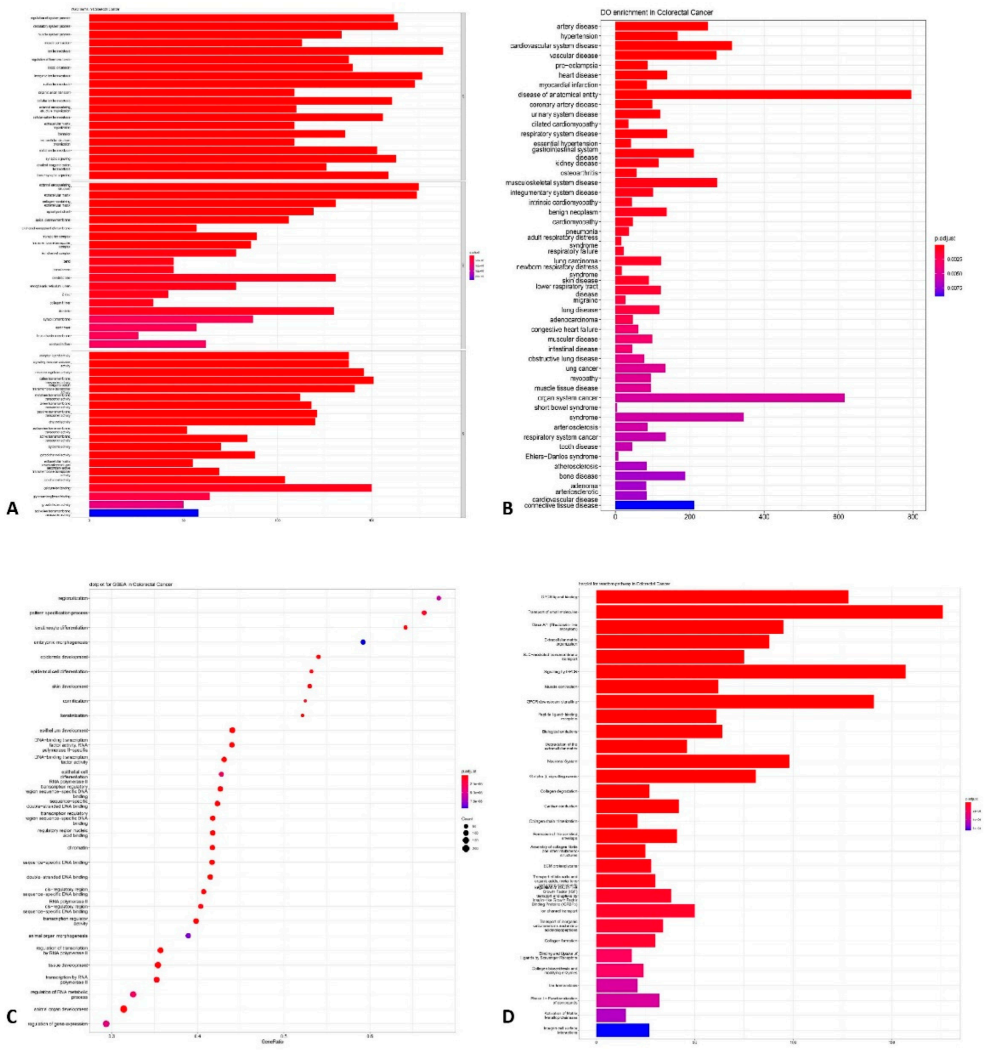

Figure S5. Pathway enrichment analyses of DEGs in Receiving targeted therapies CRC patients from the TCGA database. (A: Bar plot of Gene Ontology, B: Bar plot of Disease Ontology, C: Dot plot of gene set enrichment analysis, D: Bar plot of Reactom)

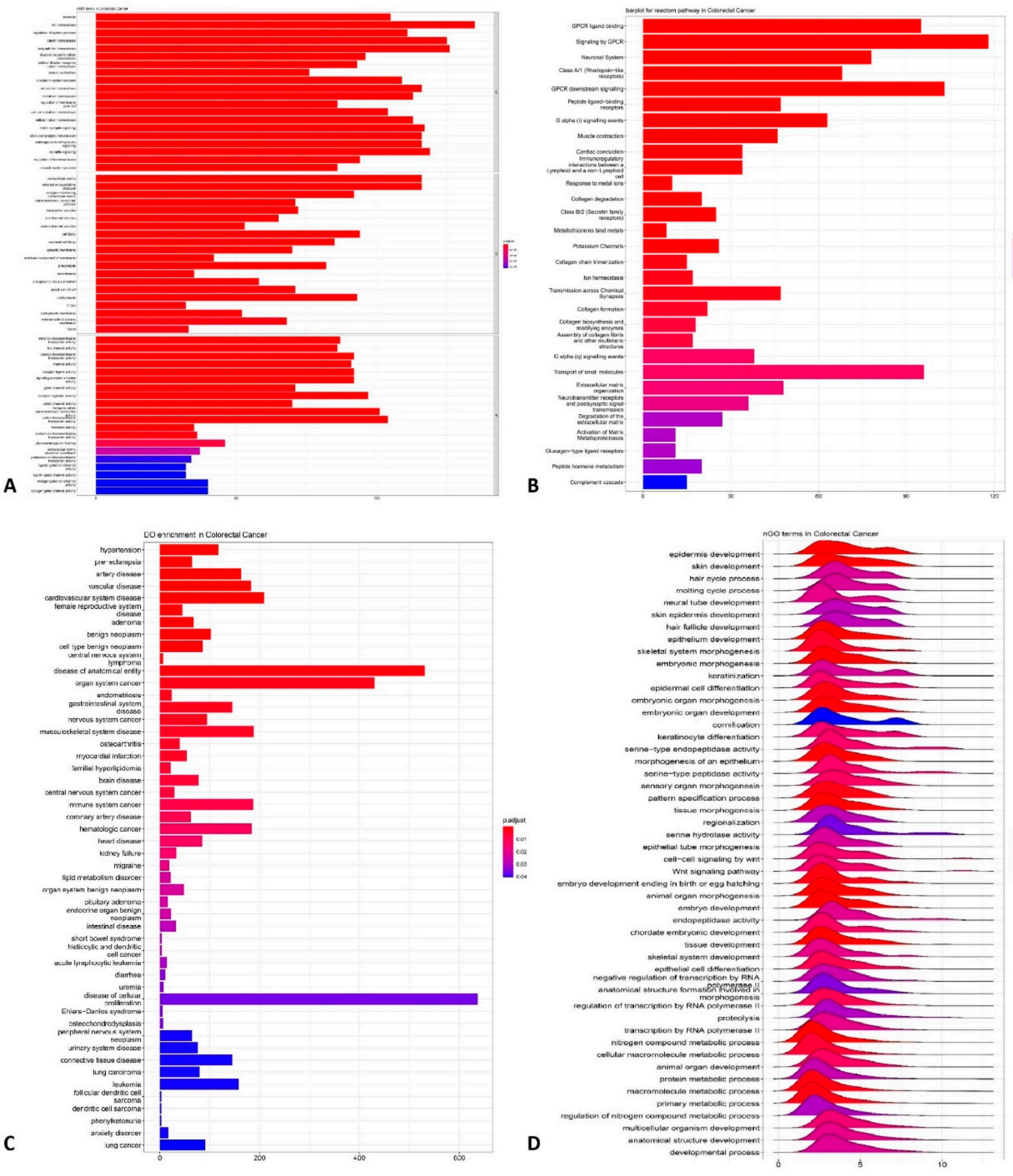

Figure S6. Pathway enrichment analyses of DEGs in Receiving chemotherapies CRC patients from the TCGA database. (A: Bar plot of Gene Ontology, B: Bar plot of Disease Ontology, C and D: Dot plots of GSEA, E: Ridgeplot for gene set enrichment analysis)

A

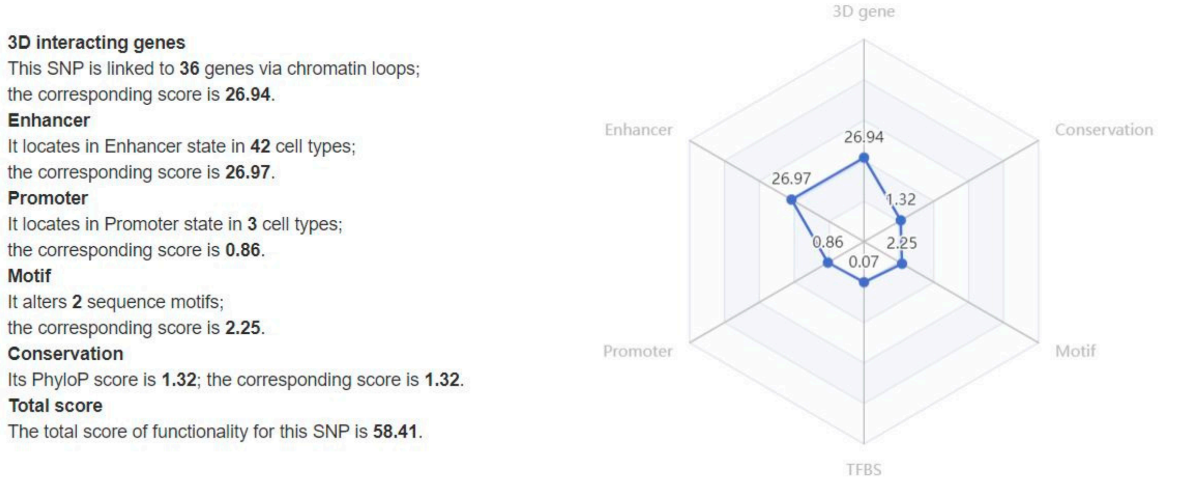

B

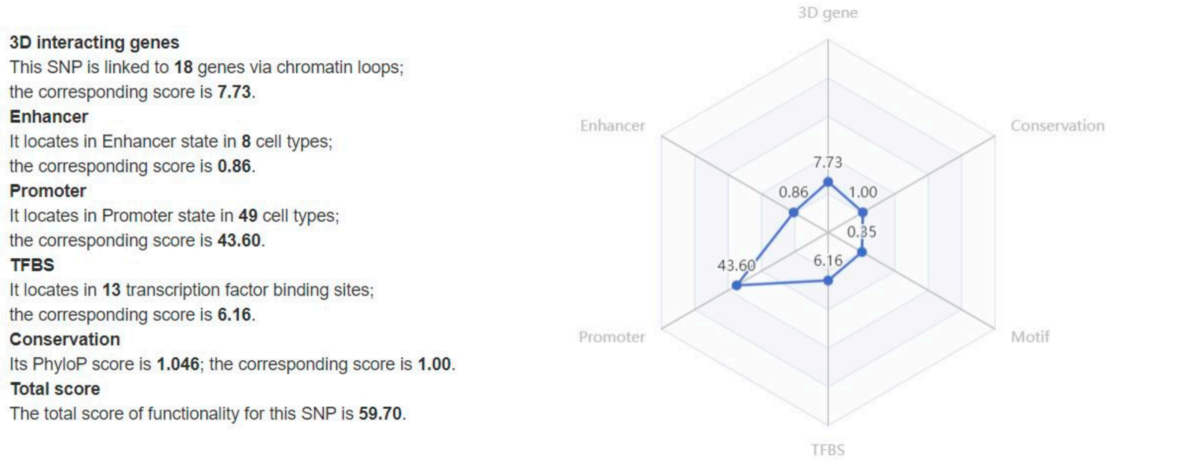

C

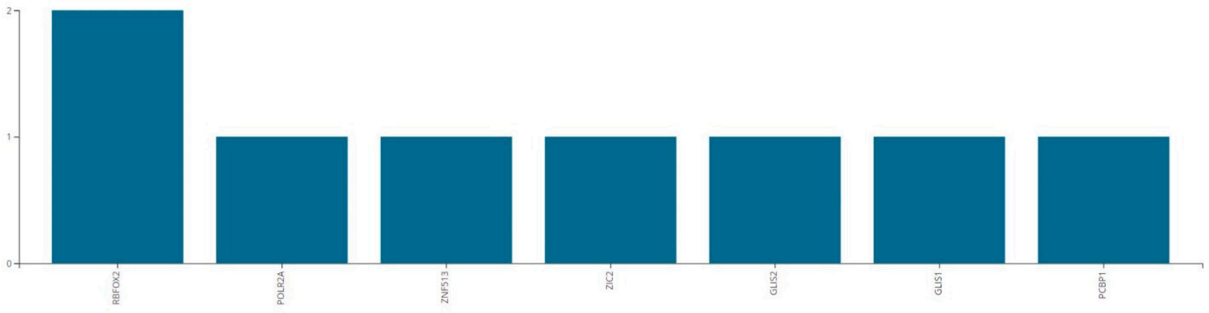

Figure S7. Results of RegulomeDB and 3DSNP database. (A: rs1428982750 variant of ZBTB12 gene in 3DSNP database, B: rs925939730 variant of the ASPHD1 gene in 3DSNP database , C: chromatin immunoprecipitation coupled to sequencing (CHIP-seq) results of rs1428982750 variant of ZBTB12 gene in RegulomeDB database.

Table S1. Number of DEGs in patients with CRC

	Number of DEGs	DEGs after Correction
**Stage I and II**		
**MSI-L**	*Increased*: 1252<br>*Decreased*: 2102	3354
**MSI-H**	*Increased*: 1246<br>*Decreased*: 1908	3154
**MSI-S**	*Increased*: 1151<br>*Decreased*: 1992	3143
**Stage III and IV**		
**Receiving chemotherapies**	*Increased*: 1336<br>*Decreased*: 1420	2757
**Receiving targeted therapies**	*Increased*: 775<br>*Decreased*: 1022	1798

Table S2. Number of survival-related DEGs.

Subgroup	Number of Survival-Related Genes
**MSI-L**	0
**MSI-H**	357
**MSI-S**	100
**Receiving chemotherapies**	38
**Receiving targeted therapies**	8

Table S3. Candidate genes based on database search

Gene	Subgroup	log FC	Patients (%) Possessing Gene Expression
**ASPHD1**	MSI-H/MSI-S	2.6	100%
**ZBTB12**	MSI-S	1.55	100%
**ONECUT3**	MSI-H<br>(early stages)	8.6	62%
**C2orf61**	MSI-H<br>(early stages)	3.1	81%
**MIA**	MSI-H<br>(early stages)	2.7	100%
**VRTN**	MSI-H<br>(early stages)	2.6	81%
**NEK5**	MSI-H<br>(early stages)	2.6	96%
**ASPHD1**	MSI-H/MSS<br>(early stages)	2.6	100%
**TMEM145**	MSI-H/MSS<br>(early stages)	2.3	100%
**CCDC150**	MSI-H<br>(early stages)	2.2	100%
**SPDYC**	MSS<br>(early stages)	3.9	88%
**C6orf223**	MSS<br>(early stages)	4.6	100%
**ZBTB12**	MSS<br>(early stages)	1.55	100%
**PNPLA3**	Conventional (advance stages)	3.1	100%
**CADPS**	Conventional (advance stages)	2.8	100%
**DCAF4L1**	Conventional (advance stages)	1.5	90%

Table S4. Variants of candidate genes in Whole Exome Sequencing (WES) data of 15 patients with CRC in Mashhad papulation.

Pateint ID	Chr	Start	End	Ref	Alt	Zygosity	Func.refGe	Gene.refG	GeneDetai	ExonicFunc.refGene	AAChange.refGene	snp138
L25.csv.18	chr2	1.98E+08	1.98E+08	T	G	het	exonic	CCDC150	.	nonsynonymous SNV	CCDC150:NM_001080539:exon18:c.T1951G:p.L651V	.
L25.csv.37	chr3	62578336	62578336	C	T	het	exonic	CADPS	.	synonymous SNV	CADPS:NM_003716:exon7:c.G1413A:p.A471A,CADPS:NM_183393:exon7:c.G1413A:p.A471A,CADPS:NM_183394:exon7:c.G1413A:p.A471A	rs17066673
L25.csv.78	chr6	43968814	43968814	A	G	het	exonic	C6orf223	.	nonsynonymous SNV	C6orf223:NM_001171992:exon2:c.A185G:p.Q62R,C6orf223:NM_153246:exon2:c.A142G:p.K48E	rs2295333
L25.csv.79	chr6	43970503	43970503	-	GCGGCG	het	exonic	C6orf223	.	nonframeshift insertion	C6orf223:NM_153246:exon4:c.369_370insGCGGCG:p.R123delinsRAA	.
L25.csv.80	chr6	43970827	43970827	G	A	het	exonic	C6orf223	.	synonymous SNV	C6orf223:NM_153246:exon4:c.G693A:p.A231A	rs2295334
L25.csv.90	chr13	52676275	52676275	T	G	het	exonic	NEK5	.	nonsynonymous SNV	NEK5:NM_199289:exon10:c.A763C:p.K255Q	rs34756139
L25.csv.91	chr13	52693493	52693493	-	TCA	het	exonic	NEK5	.	nonframeshift insertion	NEK5:NM_199289:exon4:c.175_176insTGA:p.K59delinsMK	.
L25.csv.99	chr16	29912802	29912802	-	GGT	het	exonic	ASPHD1	.	nonframeshift insertion	ASPHD1:NM_181718:exon1:c.510_511insGGT:p.Q170delinsQG	rs140411458
L25.csv.106	chr22	44324727	44324727	C	G	het	exonic	PNPLA3	.	nonsynonymous SNV	PNPLA3:NM_025225:exon3:c.C444G:p.I148M	rs738409
L25.csv.107	chr22	44324730	44324730	C	T	het	exonic	PNPLA3	.	synonymous SNV	PNPLA3:NM_025225:exon3:c.C447T:p.P149P	rs738408
L25.csv.110	chr22	44328832	44328832	C	T	het	exonic	PNPLA3	.	synonymous SNV	PNPLA3:NM_025225:exon4:c.C561T:p.F187F	rs34179073
L25.csv.123	chr22	44342116	44342116	A	G	hom	exonic	PNPLA3	.	nonsynonymous SNV	PNPLA3:NM_025225:exon9:c.A1300G:p.K434E	rs2294918
L32.csv.21	chr2	1.98E+08	1.98E+08	TG	-	het	exonic	CCDC150	.	frameshift deletion	CCDC150:NM_001080539:exon3:c.289_290del:p.C97fs	rs143904259
L32.csv.28	chr2	1.98E+08	1.98E+08	G	A	het	exonic	CCDC150	.	synonymous SNV	CCDC150:NM_001080539:exon12:c.G1281A:p.E427E	rs17271281
L32.csv.51	chr3	62543123	62543123	T	C	het	exonic	CADPS	.	synonymous SNV	CADPS:NM_003716:exon10:c.A1710G:p.L570L,CADPS:NM_183393:exon10:c.A1710G:p.L570L,CADPS:NM_183394:exon10:c.A1710G:p.L570L	rs4396888
L32.csv.103	chr4	41984118	41984118	C	T	het	exonic	DCAF4L1	.	synonymous SNV	DCAF4L1:NM_001029955:exon1:c.C309T:p.S103S	rs2660320
L32.csv.120	chr16	29912802	29912802	-	GGT	het	exonic	ASPHD1	.	nonframeshift insertion	ASPHD1:NM_181718:exon1:c.510_511insGGT:p.Q170delinsQG	rs140411458
L32.csv.128	chr22	44322922	44322922	T	G	het	exonic	PNPLA3	.	nonsynonymous SNV	PNPLA3:NM_025225:exon2:c.T295G:p.C99G	rs2076213
L32.csv.131	chr22	44324727	44324727	C	G	het	exonic	PNPLA3	.	nonsynonymous SNV	PNPLA3:NM_025225:exon3:c.C444G:p.I148M	rs738409
L32.csv.132	chr22	44324730	44324730	C	T	het	exonic	PNPLA3	.	synonymous SNV	PNPLA3:NM_025225:exon3:c.C447T:p.P149P	rs738408
L32.csv.151	chr22	44342116	44342116	A	G	hom	exonic	PNPLA3	.	nonsynonymous SNV	PNPLA3:NM_025225:exon9:c.A1300G:p.K434E	rs2294918
L33.csv.15	chr2	47382342	47382342	G	T	het	exonic	C2orf61	.	nonsynonymous SNV	C2orf61:NM_001163561:exon1:c.C49A:p.L17M,C2orf61:NM_173649:exon1:c.C49A:p.L17M	rs815804
L33.csv.108	chr4	41984118	41984118	C	T	hom	exonic	DCAF4L1	.	synonymous SNV	DCAF4L1:NM_001029955:exon1:c.C309T:p.S103S	rs2660320
L33.csv.110	chr6	43970503	43970503	-	GCG	het	exonic	C6orf223	.	nonframeshift insertion	C6orf223:NM_153246:exon4:c.369_370insGCG:p.R123delinsRA	.
L33.csv.132	chr16	29912802	29912802	-	GGT	het	exonic	ASPHD1	.	nonframeshift insertion	ASPHD1:NM_181718:exon1:c.510_511insGGT:p.Q170delinsQG	rs140411458
L33.csv.138	chr22	44324727	44324727	C	G	hom	exonic	PNPLA3	.	nonsynonymous SNV	PNPLA3:NM_025225:exon3:c.C444G:p.I148M	rs738409
L33.csv.139	chr22	44324730	44324730	C	T	hom	exonic	PNPLA3	.	synonymous SNV	PNPLA3:NM_025225:exon3:c.C447T:p.P149P	rs738408
L33.csv.156	chr22	44342116	44342116	A	G	het	exonic	PNPLA3	.	nonsynonymous SNV	PNPLA3:NM_025225:exon9:c.A1300G:p.K434E	rs2294918
L34.csv.20	chr2	47382342	47382342	G	T	het	exonic	C2orf61	.	nonsynonymous SNV	C2orf61:NM_001163561:exon1:c.C49A:p.L17M,C2orf61:NM_173649:exon1:c.C49A:p.L17M	rs815804
L34.csv.24	chr2	1.98E+08	1.98E+08	TG	-	het	exonic	CCDC150	.	frameshift deletion	CCDC150:NM_001080539:exon3:c.289_290del:p.C97fs	rs143904259
L34.csv.48	chr3	62543123	62543123	T	C	het	exonic	CADPS	.	synonymous SNV	CADPS:NM_003716:exon10:c.A1710G:p.L570L,CADPS:NM_183393:exon10:c.A1710G:p.L570L,CADPS:NM_183394:exon10:c.A1710G:p.L570L	rs4396888
L34.csv.88	chr4	41984118	41984118	C	T	het	exonic	DCAF4L1	.	synonymous SNV	DCAF4L1:NM_001029955:exon1:c.C309T:p.S103S	rs2660320
L34.csv.106	chr16	29912802	29912802	-	GGT	hom	exonic	ASPHD1	.	nonframeshift insertion	ASPHD1:NM_181718:exon1:c.510_511insGGT:p.Q170delinsQG	rs140411458
L34.csv.111	chr19	41281700	41281700	C	T	het	exonic	MIA	.	synonymous SNV	MIA:NM_006533:exon2:c.C171T:p.P57P,MIA:NM_001202553:exon3:c.C171T:p.P57P	rs2233156
L34.csv.113	chr22	44328832	44328832	C	T	hom	exonic	PNPLA3	.	synonymous SNV	PNPLA3:NM_025225:exon4:c.C561T:p.F187F	rs34179073
L34.csv.117	chr22	44342116	44342116	A	G	hom	exonic	PNPLA3	.	nonsynonymous SNV	PNPLA3:NM_025225:exon9:c.A1300G:p.K434E	rs2294918
L44.csv.58	chr3	62578336	62578336	C	T	het	exonic	CADPS	.	synonymous SNV	CADPS:NM_003716:exon7:c.G1413A:p.A471A,CADPS:NM_183393:exon7:c.G1413A:p.A471A,CADPS:NM_183394:exon7:c.G1413A:p.A471A	rs17066673
L44.csv.104	chr4	41984118	41984118	C	T	hom	exonic	DCAF4L1	.	synonymous SNV	DCAF4L1:NM_001029955:exon1:c.C309T:p.S103S	rs2660320
L44.csv.107	chr6	43968814	43968814	A	G	het	exonic	C6orf223	.	nonsynonymous SNV	C6orf223:NM_001171992:exon2:c.A185G:p.Q62R,C6orf223:NM_153246:exon2:c.A142G:p.K48E	rs2295333
L44.csv.108	chr6	43970503	43970503	-	GCG	het	exonic	C6orf223	.	nonframeshift insertion	C6orf223:NM_153246:exon4:c.369_370insGCG:p.R123delinsRA	.
L44.csv.109	chr6	43970503	43970503	-	GCGGCG	het	exonic	C6orf223	.	nonframeshift insertion	C6orf223:NM_153246:exon4:c.369_370insGCGGCG:p.R123delinsRAA	.
L44.csv.110	chr6	43970827	43970827	G	A	het	exonic	C6orf223	.	synonymous SNV	C6orf223:NM_153246:exon4:c.G693A:p.A231A	rs2295334
L44.csv.134	chr16	29912802	29912802	-	GGT	hom	exonic	ASPHD1	.	nonframeshift insertion	ASPHD1:NM_181718:exon1:c.510_511insGGT:p.Q170delinsQG	rs140411458
L44.csv.141	chr22	44322922	44322922	T	G	het	exonic	PNPLA3	.	nonsynonymous SNV	PNPLA3:NM_025225:exon2:c.T295G:p.C99G	rs2076213
L44.csv.143	chr22	44324727	44324727	C	G	hom	exonic	PNPLA3	.	nonsynonymous SNV	PNPLA3:NM_025225:exon3:c.C444G:p.I148M	rs738409
L44.csv.144	chr22	44324730	44324730	C	T	hom	exonic	PNPLA3	.	synonymous SNV	PNPLA3:NM_025225:exon3:c.C447T:p.P149P	rs738408
L44.csv.169	chr22	44342116	44342116	A	G	hom	exonic	PNPLA3	.	nonsynonymous SNV	PNPLA3:NM_025225:exon9:c.A1300G:p.K434E	rs2294918
L5.csv.50	chr3	62467521	62467521	T	A	het	exonic	CADPS	.	nonsynonymous SNV	CADPS:NM_003716:exon22:c.A3050T:p.N1017I	rs77008736
L5.csv.127	chr4	41984118	41984118	C	T	hom	exonic	DCAF4L1	.	synonymous SNV	DCAF4L1:NM_001029955:exon1:c.C309T:p.S103S	rs2660320
L5.csv.156	chr16	29912802	29912802	-	GGT	het	exonic	ASPHD1	.	nonframeshift insertion	ASPHD1:NM_181718:exon1:c.510_511insGGT:p.Q170delinsQG	rs140411458
L5.csv.172	chr22	44324727	44324727	C	G	het	exonic	PNPLA3	.	nonsynonymous SNV	PNPLA3:NM_025225:exon3:c.C444G:p.I148M	rs738409
L5.csv.173	chr22	44324730	44324730	C	T	het	exonic	PNPLA3	.	synonymous SNV	PNPLA3:NM_025225:exon3:c.C447T:p.P149P	rs738408
L5.csv.180	chr22	44342116	44342116	A	G	hom	exonic	PNPLA3	.	nonsynonymous SNV	PNPLA3:NM_025225:exon9:c.A1300G:p.K434E	rs2294918
L50.csv.95	chr4	41984118	41984118	C	T	het	exonic	DCAF4L1	.	synonymous SNV	DCAF4L1:NM_001029955:exon1:c.C309T:p.S103S	rs2660320
L50.csv.121	chr16	29912802	29912802	-	GGT	het	exonic	ASPHD1	.	nonframeshift insertion	ASPHD1:NM_181718:exon1:c.510_511insGGT:p.Q170delinsQG	rs140411458
L50.csv.132	chr19	41281700	41281700	C	T	het	exonic	MIA	.	synonymous SNV	MIA:NM_006533:exon2:c.C171T:p.P57P,MIA:NM_001202553:exon3:c.C171T:p.P57P	rs2233156
L50.csv.139	chr22	44324727	44324727	C	G	het	exonic	PNPLA3	.	nonsynonymous SNV	PNPLA3:NM_025225:exon3:c.C444G:p.I148M	rs738409
L50.csv.140	chr22	44324730	44324730	C	T	het	exonic	PNPLA3	.	synonymous SNV	PNPLA3:NM_025225:exon3:c.C447T:p.P149P	rs738408
L50.csv.160	chr22	44342116	44342116	A	G	het	exonic	PNPLA3	.	nonsynonymous SNV	PNPLA3:NM_025225:exon9:c.A1300G:p.K434E	rs2294918
L51.csv.34	chr2	47382342	47382342	G	T	het	exonic	C2orf61	.	nonsynonymous SNV	C2orf61:NM_001163561:exon1:c.C49A:p.L17M,C2orf61:NM_173649:exon1:c.C49A:p.L17M	rs815804
L51.csv.181	chr4	41984118	41984118	C	T	hom	exonic	DCAF4L1	.	synonymous SNV	DCAF4L1:NM_001029955:exon1:c.C309T:p.S103S	rs2660320
L51.csv.212	chr19	1754433	1754433	G	T	hom	exonic	ONECUT3	.	nonsynonymous SNV	ONECUT3:NM_001080488:exon1:c.G772T:p.A258S	.
L51.csv.225	chr22	44324727	44324727	C	G	het	exonic	PNPLA3	.	nonsynonymous SNV	PNPLA3:NM_025225:exon3:c.C444G:p.I148M	rs738409
L51.csv.226	chr22	44324730	44324730	C	T	het	exonic	PNPLA3	.	synonymous SNV	PNPLA3:NM_025225:exon3:c.C447T:p.P149P	rs738408
L51.csv.240	chr22	44342116	44342116	A	G	hom	exonic	PNPLA3	.	nonsynonymous SNV	PNPLA3:NM_025225:exon9:c.A1300G:p.K434E	rs2294918
L52.csv.24	chr2	47382342	47382342	G	T	het	exonic	C2orf61	.	nonsynonymous SNV	C2orf61:NM_001163561:exon1:c.C49A:p.L17M,C2orf61:NM_173649:exon1:c.C49A:p.L17M	rs815804
L52.csv.163	chr4	41984118	41984118	C	T	hom	exonic	DCAF4L1	.	synonymous SNV	DCAF4L1:NM_001029955:exon1:c.C309T:p.S103S	rs2660320
L52.csv.176	chr13	52676275	52676275	T	G	het	exonic	NEK5	.	nonsynonymous SNV	NEK5:NM_199289:exon10:c.A763C:p.K255Q	rs34756139
L52.csv.179	chr16	29912802	29912802	-	GGT	hom	exonic	ASPHD1	.	nonframeshift insertion	ASPHD1:NM_181718:exon1:c.510_511insGGT:p.Q170delinsQG	rs140411458
L52.csv.183	chr19	42817572	42817572	C	T	het	exonic	TMEM145	.	nonsynonymous SNV	TMEM145:NM_173633:exon1:c.C44T:p.P15L	rs185966365
L52.csv.186	chr19	42827940	42827940	C	T	het	exonic	TMEM145	.	nonsynonymous SNV	TMEM145:NM_173633:exon14:c.C1400T:p.S467F	rs141686253
L52.csv.190	chr22	44324727	44324727	C	G	het	exonic	PNPLA3	.	nonsynonymous SNV	PNPLA3:NM_025225:exon3:c.C444G:p.I148M	rs738409
L52.csv.191	chr22	44324730	44324730	C	T	het	exonic	PNPLA3	.	synonymous SNV	PNPLA3:NM_025225:exon3:c.C447T:p.P149P	rs738408
L52.csv.214	chr22	44342116	44342116	A	G	het	exonic	PNPLA3	.	nonsynonymous SNV	PNPLA3:NM_025225:exon9:c.A1300G:p.K434E	rs2294918
L54.csv.156	chr4	41984118	41984118	C	T	hom	exonic	DCAF4L1	.	synonymous SNV	DCAF4L1:NM_001029955:exon1:c.C309T:p.S103S	rs2660320
L54.csv.170	chr13	52667228	52667228	G	A	het	exonic	NEK5	.	synonymous SNV	NEK5:NM_199289:exon13:c.C1170T:p.Y390Y	rs55715265
L54.csv.195	chr22	44322922	44322922	T	G	het	exonic	PNPLA3	.	nonsynonymous SNV	PNPLA3:NM_025225:exon2:c.T295G:p.C99G	rs2076213
L54.csv.196	chr22	44322970	44322970	G	T	het	exonic	PNPLA3	.	nonsynonymous SNV	PNPLA3:NM_025225:exon2:c.G343T:p.G115C	rs2076212
L59.csv.28	chr2	47382342	47382342	G	T	het	exonic	C2orf61	.	nonsynonymous SNV	C2orf61:NM_001163561:exon1:c.C49A:p.L17M,C2orf61:NM_173649:exon1:c.C49A:p.L17M	rs815804
L59.csv.31	chr2	1.98E+08	1.98E+08	G	A	het	exonic	CCDC150	.	synonymous SNV	CCDC150:NM_001080539:exon12:c.G1281A:p.E427E	rs17271281
L59.csv.157	chr4	41984118	41984118	C	T	het	exonic	DCAF4L1	.	synonymous SNV	DCAF4L1:NM_001029955:exon1:c.C309T:p.S103S	rs2660320
L59.csv.159	chr6	43968814	43968814	A	G	het	exonic	C6orf223	.	nonsynonymous SNV	C6orf223:NM_001171992:exon2:c.A185G:p.Q62R,C6orf223:NM_153246:exon2:c.A142G:p.K48E	rs2295333
L59.csv.160	chr6	43970503	43970503	-	GCGGCG	het	exonic	C6orf223	.	nonframeshift insertion	C6orf223:NM_153246:exon4:c.369_370insGCGGCG:p.R123delinsRAA	.
L59.csv.161	chr6	43970827	43970827	G	A	het	exonic	C6orf223	.	synonymous SNV	C6orf223:NM_153246:exon4:c.G693A:p.A231A	rs2295334
L59.csv.180	chr16	29912802	29912802	-	GGT	hom	exonic	ASPHD1	.	nonframeshift insertion	ASPHD1:NM_181718:exon1:c.510_511insGGT:p.Q170delinsQG	rs140411458
L59.csv.184	chr19	1754615	1754615	C	T	het	exonic	ONECUT3	.	synonymous SNV	ONECUT3:NM_001080488:exon1:c.C954T:p.A318A	rs370471416
L59.csv.200	chr22	44322970	44322970	G	T	het	exonic	PNPLA3	.	nonsynonymous SNV	PNPLA3:NM_025225:exon2:c.G343T:p.G115C	rs2076212
L59.csv.207	chr22	44342116	44342116	A	G	het	exonic	PNPLA3	.	nonsynonymous SNV	PNPLA3:NM_025225:exon9:c.A1300G:p.K434E	rs2294918
L6.csv.19	chr2	1.98E+08	1.98E+08	G	A	het	exonic	CCDC150	.	synonymous SNV	CCDC150:NM_001080539:exon12:c.G1281A:p.E427E	rs17271281
L6.csv.86	chr4	41984118	41984118	C	T	hom	exonic	DCAF4L1	.	synonymous SNV	DCAF4L1:NM_001029955:exon1:c.C309T:p.S103S	rs2660320
L6.csv.108	chr16	29912802	29912802	-	GGT	het	exonic	ASPHD1	.	nonframeshift insertion	ASPHD1:NM_181718:exon1:c.510_511insGGT:p.Q170delinsQG	rs140411458
L6.csv.116	chr22	44328832	44328832	C	T	het	exonic	PNPLA3	.	synonymous SNV	PNPLA3:NM_025225:exon4:c.C561T:p.F187F	rs34179073
L6.csv.121	chr22	44342116	44342116	A	G	hom	exonic	PNPLA3	.	nonsynonymous SNV	PNPLA3:NM_025225:exon9:c.A1300G:p.K434E	rs2294918
L7.csv.58	chr3	62578336	62578336	C	T	het	exonic	CADPS	.	synonymous SNV	CADPS:NM_003716:exon7:c.G1413A:p.A471A,CADPS:NM_183393:exon7:c.G1413A:p.A471A,CADPS:NM_183394:exon7:c.G1413A:p.A471A	rs17066673
L7.csv.109	chr4	41984118	41984118	C	T	hom	exonic	DCAF4L1	.	synonymous SNV	DCAF4L1:NM_001029955:exon1:c.C309T:p.S103S	rs2660320
L7.csv.124	chr16	29912802	29912802	-	GGT	het	exonic	ASPHD1	.	nonframeshift insertion	ASPHD1:NM_181718:exon1:c.510_511insGGT:p.Q170delinsQG	rs140411458
L7.csv.125	chr22	44322922	44322922	T	G	het	exonic	PNPLA3	.	nonsynonymous SNV	PNPLA3:NM_025225:exon2:c.T295G:p.C99G	rs2076213
L7.csv.128	chr22	44324727	44324727	C	G	hom	exonic	PNPLA3	.	nonsynonymous SNV	PNPLA3:NM_025225:exon3:c.C444G:p.I148M	rs738409
L7.csv.129	chr22	44324730	44324730	C	T	hom	exonic	PNPLA3	.	synonymous SNV	PNPLA3:NM_025225:exon3:c.C447T:p.P149P	rs738408
L7.csv.153	chr22	44342116	44342116	A	G	hom	exonic	PNPLA3	.	nonsynonymous SNV	PNPLA3:NM_025225:exon9:c.A1300G:p.K434E	rs2294918
L8.csv.28	chr2	47382342	47382342	G	T	het	exonic	C2orf61	.	nonsynonymous SNV	C2orf61:NM_001163561:exon1:c.C49A:p.L17M,C2orf61:NM_173649:exon1:c.C49A:p.L17M	rs815804
L8.csv.31	chr2	1.98E+08	1.98E+08	G	A	het	exonic	CCDC150	.	nonsynonymous SNV	CCDC150:NM_001080539:exon4:c.G466A:p.E156K	rs34133636
L8.csv.34	chr2	1.98E+08	1.98E+08	G	A	het	exonic	CCDC150	.	synonymous SNV	CCDC150:NM_001080539:exon12:c.G1281A:p.E427E	rs17271281
L8.csv.135	chr4	41984118	41984118	C	T	het	exonic	DCAF4L1	.	synonymous SNV	DCAF4L1:NM_001029955:exon1:c.C309T:p.S103S	rs2660320
L8.csv.160	chr19	1754183	1754183	G	A	het	exonic	ONECUT3	.	synonymous SNV	ONECUT3:NM_001080488:exon1:c.G522A:p.E174E	.
L8.csv.180	chr22	44342116	44342116	A	G	het	exonic	PNPLA3	.	nonsynonymous SNV	PNPLA3:NM_025225:exon9:c.A1300G:p.K434E	rs2294918
L9.csv.47	chr3	62578336	62578336	C	T	het	exonic	CADPS	.	synonymous SNV	CADPS:NM_003716:exon7:c.G1413A:p.A471A,CADPS:NM_183393:exon7:c.G1413A:p.A471A,CADPS:NM_183394:exon7:c.G1413A:p.A471A	rs17066673
L9.csv.88	chr4	41984118	41984118	C	T	hom	exonic	DCAF4L1	.	synonymous SNV	DCAF4L1:NM_001029955:exon1:c.C309T:p.S103S	rs2660320
L9.csv.100	chr19	1753933	1753933	C	A	het	exonic	ONECUT3	.	nonsynonymous SNV	ONECUT3:NM_001080488:exon1:c.C272A:p.A91E	.

Table S5. The results of the effect of rs1428982750 variant of ZBTB12 gene on chromatin status in colon and rectum.

Chromatin State	Chromatin State Window	Biosample	Organ
**Strong transcription**	chr6:31866800..31868800	sigmoid colon	intestine, colon, large intestine
**Strong transcription**	chr6:31867600..31868600	muscle layer of colon	intestine, colon, large intestine
**Strong transcription**	chr6:31866800..31868800	mucosa of rectum	intestine, large intestine
**Strong transcription**	chr6:31866800..31868600	colonic mucosa	intestine, colon, large intestine
**Strong transcription**	chr6:31866800..31868800	Rectal smooth muscle tissue	intestine, musculature of body, large intestine
**Strong transcription**	chr6:31867200..31868600	large intestine	intestine, large intestine
**Strong transcription**	chr6:31867000..31868800	mucosa of rectum	intestine, large intestine

Table S6. The results of the effect of rs925939730 variant of ASPHD1 gene on chromatin status in colon and rectum.

CHROMATIN STATE	Chromatin State window	Biosample	Organ
**Repressed PolyComb**	chr16:29912600..29917200	Rectal smooth muscle tissue	intestine, musculature of body, large intestine
**Repressed PolyComb**	chr16:29912400..29913000	muscle layer of colon	intestine, colon, large intestine
**Strong transcription**	chr16:29912200..29930400	mucosa of rectum	intestine, large intestine
**Strong transcription**	chr16:29912200..29925600	mucosa of rectum	intestine, large intestine
**Strong transcription**	chr16:29912200..29918000	colonic mucosa	intestine, colon, large intestine
**Strong transcription**	chr16:29912000..29924800	sigmoid colon	intestine, colon, large intestine

Table S7. Results of rs1428982750 variant of ZBTB12 gene in 3DSNP database

Variant ID	Position	Score	3D interacting Gene	Enhancer	Promoter	TFBS	Motif
**rs1428982750**	chr6:31868322	58.4	VARS and other 35	42	3	0	2

Table S8. Results of rs925939730 variant of ASPHD1 gene in 3DSNP database

Variant ID	Position	Score	3D Interacting Gene	Enhancer	Promoter	TFBS
**rs925939730**	chr16:29912659	59.7	MAZ and other 17	8	49	13
